# Identification of genetic association between mitochondrial dysfunction and knee osteoarthritis through integrating multi-omics: a summary data-based Mendelian randomization study

**DOI:** 10.1007/s10067-024-07136-7

**Published:** 2024-09-11

**Authors:** Jiale Xie, Rui Ma, Xin Xu, Mingyi Yang, Hui Yu, Xianjie Wan, Ke Xu, Junfei Guo, Peng Xu

**Affiliations:** Department of Joint Surgery, HongHui Hospital, Xian Jiaotong University, Xian, Shaanxi China

**Keywords:** Bayesian colocalization analysis, Knee osteoarthritis, Mendelian randomization, Mitochondrial dysfunction, Quantitative trait loci, Therapeutic targets

## Abstract

**Abstract:**

**Objective:**

Association between mitochondrial dysfunction and osteoarthritis (OA) has been consistently investigated, yet their genetic association remains obscure. In this study, mitochondrial-related genes were used as instrumental variables to proxy for mitochondrial dysfunction, and summary data of knee OA (KOA) were used as outcome to examine their genetic association.

**Methods:**

We obtained 1136 mitochondrial-related genes from the human MitoCarta3.0 database. Genetic proxy instruments for mitochondrial-related genes from studies of corresponding gene expression (*n* = 31,684) and protein (*n* = 35,559) quantitative trait locus (eQTLs and pQTLs), respectively. Aggregated data for KOA (62,497 KOA cases and 333,557 controls) were extracted from the largest OA genome-wide association study (GWAS). We integrated QTL data with KOA GWAS data to estimate their genetic association using summary data-based Mendelian randomization analysis (SMR). Additionally, we implemented Bayesian colocalization analysis to reveal whether suggestive mitochondrial-related genes and KOA were driven by a same genetic variant. Finally, to validate the primary findings, replication study (24,955 cases and 378,169 controls) and multi-SNP-based SMR (SMR-multi) test was performed.

**Results:**

Through SMR analysis, we found that the expression levels of 2 mitochondrial-related genes were associated with KOA risk. Specifically, elevated gene expression levels of the IMMP2L (odds ratio [OR] = 1.056; 95% confidence interval [CI] = 1.030–1.082; *P-*_*FDR*_ = 0.004) increased the risk of KOA. Conversely, increased gene expression levels of AKAP10 decreased the risk of KOA (OR = 0.955; 95% CI, 0.934–0.977; *P-*_*FDR*_ = 0.019). Colocalization analysis demonstrated that AKAP10 (PP.H4 = 0.84) and IMMP2L (PP.H4 = 0.91) shared the same genetic variant with KOA. In addition, consistent results were found in replication study and SMR-multi test, further demonstrating the reliability of our findings.

**Conclusions:**

In summary, our analyses revealed the genetic association between mitochondrial dysfunction proxied by mitochondrial-related genes and KOA, providing new insight into potential pathogenesis of KOA. Furthermore, these identified candidate genes offer the possibility of clinical drug target development for KOA.

**Table:** 

Key points	
• This is the first SMR study to explore the genetic association between mitochondrial dysfunction proxied by mitochondrial-related genes and KOA.	
• Sufficient evidence to support genetic association between the expression levels of AKAP10 and IMMP2L, and KOA	
• Our MR analysis may provide novel new insight into potential pathogenesis of KOA.	
• These identified candidate genes offer the possibility of clinical drug target development for KOA

**Supplementary Information:**

The online version contains supplementary material available at 10.1007/s10067-024-07136-7.

## Introduction

Knee osteoarthritis (KOA) is a common musculoskeletal disease among middle-aged and elderly individuals, characterized by chronic pain and functional disability [1]. It involves pathological changes in all joint tissues, including synovitis, cartilage extracellular matrix degradation, chondrocyte apoptosis, subchondral bone sclerosis, and osteophyte formation [2]. Globally, KOA accounts for approximately 80% of all OA cases. The onset of KOA symptoms peaks between the ages of 55 and 64, and the prevalence increases with age, significantly impacting patients’ quality of life and well-being [3, 4]. Although previous studies have investigated various aspects of the pathogenesis of KOA, such as abnormal mechanical load, oxidative stress, inflammatory factors, and mitochondrial dysfunction, the underlying mechanism is not fully understood [5]. Therefore, further research is necessary to deepen our understanding of the pathogenesis of KOA.

Mitochondria serve as the primary sites of energy production in eukaryotic cells and generate adenosine triphosphate (ATP) through oxidative phosphorylation. Additionally, mitochondria play crucial roles in various cellular functions, such as oxidative stress, biosynthesis and metabolism, and intercellular communication [6]. These highly dynamic cellular organelles continuously reshape their morphology through fusion and fission to adapt their functions. When mitochondrial dynamics are imbalanced, mitochondrial dysfunction occurs, leading to cell degeneration or death [7]. KOA is characterized by chondrocyte damage influenced by multiple factors, with aging closely related to its development. Mitochondrial dysfunction is an important marker of cell aging. Ageing-related mitochondrial dysfunction damages cartilage by releasing reactive oxygen species (ROS), leading to the progression of KOA [8]. During mitochondrial dysfunction, excessive ROS accumulation inhibits the oxidation of cysteine to cysteine sulfinic acid (Cys-SO2H) or sulfonic acid (Cys-SO3H), thereby accelerating disease progression by damaging cellular pathways [9]. Substantial evidence has shown that mitochondrial autophagy is involved in KOA pathogenesis. Impaired mitochondrial autophagy results in excessive ROS accumulation, contributing to KOA [10]. Jiang et al. found that Cpt1a knockout regulated the aging phenotype of chondrocytes through PINK1-mediated mitophagy [11]. Liu et al. found that alpha-ketoglutarate (alpha-KG) significantly improved mitochondrial respiration in chondrocytes, enhanced mitochondrial autophagy, and delayed articular cartilage degeneration [12]. Moreover, numerous studies have shown that mitochondrial-related genes, such as monoamine oxidase B (MAOB) and mitochondrial protein mitofusin 2 (MFN2), are related to cell aging and OA [13, 14]. In addition to the 37 key mitochondrial genes, the associated genome contained more than 1000 additional nuclear genes. The susceptibility of these genes indicated that the mitochondria are easily affected by external environmental factors [15]. Mitochondrial dysfunction is a complex process. Although the promotion of KOA by mitochondrial dysfunction has been extensively studied, the specific mechanism remains unclear, necessitating further research.

Mendelian randomization (MR) is a novel method, which uses genetic variations as instrumental variables (IVs) to offer inferences regarding the causal relationships between exposures and outcomes. Compared with randomized controlled trials (RCTs), it can effectively eliminate the interference of confounding factors and reverse causation, resulting in greatly improved accuracy and credibility of results [16]. As an extension of the MR analysis, summary data-based MR (SMR) method was developed to facilitate the inference of genetic association between diverse molecular traits (e.g., gene expression, DNA methylation, or protein abundance) and diseases of interest [17]. Compared with traditional MR analyses, SMR, using the top cis-quantitative trait loci (QTL) as the exposure, can yield higher statistical power when the exposure and outcome are obtained from two independent samples with large sample sizes [17]. Notably, in recent years, as large-scale genome-wide association study (GWAS) and molecular QTL data continue to be explored, we have opportunity to integrate them to explore susceptibility risk genes for different diseases. Up to now, limited SMR studies had identified the potential genetic association between mitochondrial dysfunction and KOA. Therefore, this study aimed to explore the genetic relationship between mitochondrial dysfunction proxied by mitochondrial-related genes and KOA using multi-omics data (including gene expression (eQTL) and protein QTL (pQTL)) data.

## Materials and methods

### Ethical considerations

Data used in our study were publicly available, and further ethical approval was not required.

### Study design

**Fig. 1:**
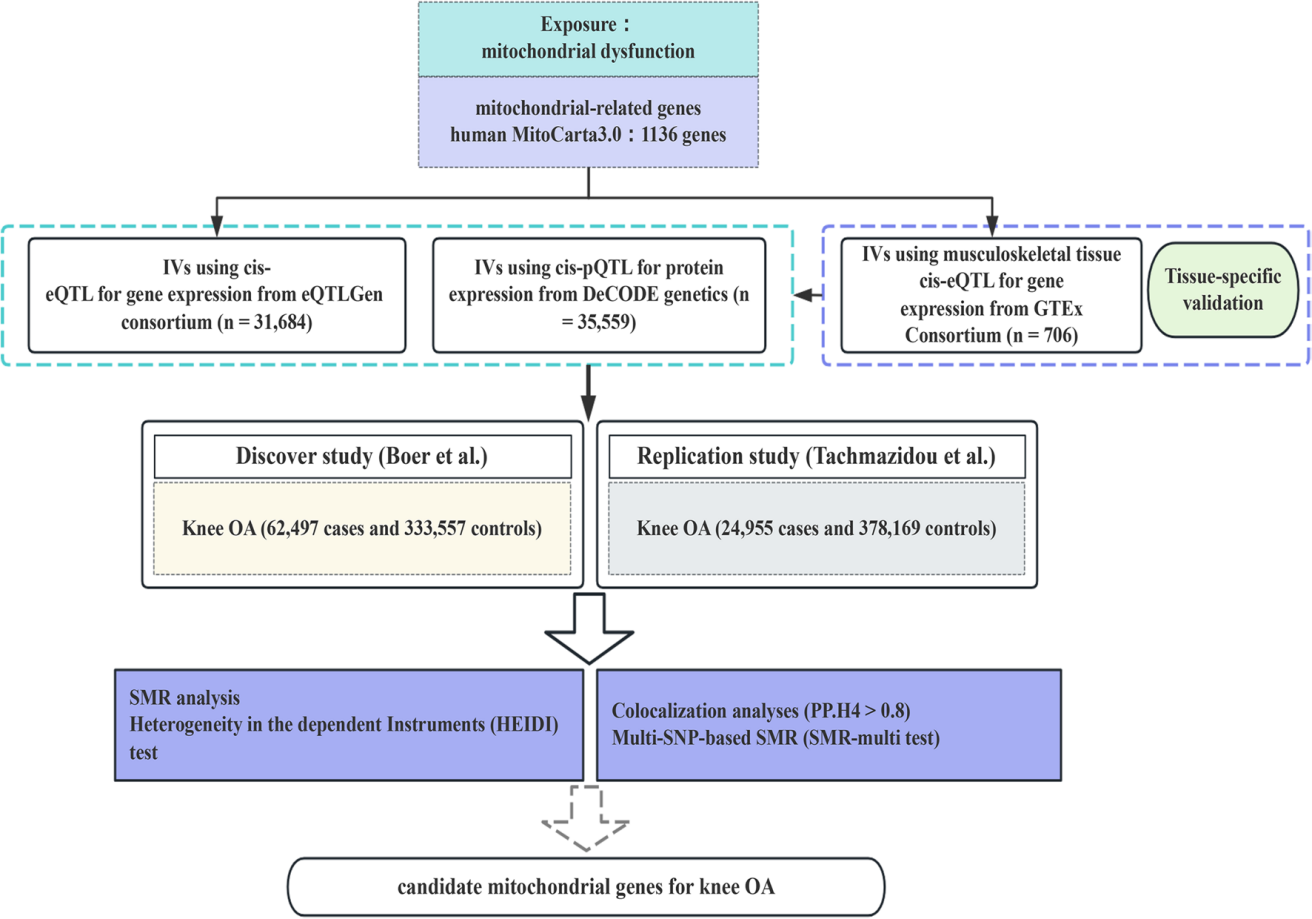
Overview of the study design. IVs, instrumental variables; QTL, quantitative trait loci; MR, Mendelian randomization; OA, osteoarthritis

Figure 1 illustrates the study design and workflow. Initially, we obtained 1,136 mitochondrial-related genes from the human MitoCarta3.0 database [18]. Then, IVs of the mitochondrial-related genes extracted from eQTLs and pQTLs were used as proxies for mitochondrial dysfunction. Their association with KOA was investigated through SMR analysis. Additionally, to improve the reliability of the results, we performed the Heterogeneity in the dependent Instruments (HEIDI) test and Bayesian colocalization analysis. Only mitochondrial-related genes that passed the HEIDI test and colocalization analysis were considered candidate genes for KOA. In present study, summarized KOA GWAS data from the Genetics of Osteoarthritis (GO) Consortium were used as the primary discovery study, and to validate our findings, summarized KOA GWAS data from the UK Biobank and Arthritis Research UK Osteoarthritis Genetics (arcOGEN) Consortium were used as the replication study. Finally, we used the multi-SNP-based SMR (SMR-multi) test to further strengthen the genetic association of the identified candidate genes with KOA.

### Data sources

The eQTL IVs of mitochondrial-related genes were sourced from the eQTLGen Consortium, which identified 16,989 cis-eQTL genes in 31,684 blood samples [19]. Gene expression probes corresponding to cis-eQTLs located within 1 Mb of the gene center distance and detected in more than one cohort (false discovery rate (FDR) < 0.05) were included (www.eqtlgen.org/cis-eqtls.html). The pQTL IVs of the mitochondrial-related genes were extracted from a large-scale proteomics-wide association study by Ferkingstad et al., which measured 4,907 plasma proteins in 35,559 individuals of European descent [20]. In the present study, cis-pQTLs that reached the *P* < 5 $\times$ 10^–8^ threshold and were within 1 Mb of their corresponding genes were selected.

Considering the tissue specificity of gene expression, we applied specific eQTL data from musculoskeletal tissues to strengthen the association of the identified candidate genes with KOA and reveal the deeper pathogenesis of KOA. Musculoskeletal tissue-specific eQTL IVs of mitochondrial-related genes were derived from the Genotype-Tissue Expression (GTEx) Consortium (*n* = 706) [21]. In this study, 49 human tissues were sequenced. Additionally, only cis-eQTLs were within 1 Mb of the transcription start site were selected.

Aggregated KOA GWAS data for discovery study were obtained from the largest GWAS meta-analysis of OA, comprising 13 international cohorts and 826,690 individuals. This study analyzed 11 common OA sites, and the summary data for KOA included 396,054 individuals (62,497 cases and 333, 557controls) [22]. To validate our findings, we used different KOA GWAS data (24,955 cases and 378,169 controls) provided by the UK Biobank and arcOGEN Consortium for the replication study [23]. More data information was presented in Supplementary Table 1.

### Statistical analysis

We used SMR analysis to explore the genetic relationship between mitochondrial dysfunction proxied by mitochondrial-related genes and KOA. In the current SMR analysis, the most significant associated top cis-QTLs of mitochondrial-related genes were selected as IVs. Notably, when specific mitochondrial-related genes had multiple most significant cis-QTLs in the QTLs data (i.e., the p-values were the same), the cis-QTL with the largest absolute value of the Z-score was selected as the top cis-QTL. Additionally, we checked the consistency of allele frequency of each SNP between pairwise datasets, including QTL data, GWAS data, and LD reference data. SNPs with allele frequency differences < 0.20 between any pair of the data sets were included. Furthermore, to minimize bias caused by pleiotropy, the HEIDI test was rationally employed. Of note, a *P*-value of HEIDI > 0.05 suggested no pleiotropy, implying that the result was reliable [24]. Finally, we used FDR correction to avoid false genetic association. Only mitochondrial-related cis-QTLs that passed FDR correction (*P* _SMR corrected by FDR < 0.05) and HEIDI test (P _HEIDI > 0.05) were allowed to undergo further colocalization analyses. Notably, we additionally obtained eQTL data from musculoskeletal tissue to validate the primary results and provide further insights into the pathogenesis of KOA. Both SMR analysis and HEIDI tests were performed using SMR software for Windows version 1.3.1.

To avoid the bias induced by weak IV, we additionally calculated the F-statistics of the IVs of the candidate mitochondrial-related genes. Of note, an F-statistic of > 10 for IV indicated that it was valid and plausible. F-statistics were calculated by the following formula: F = *R*^2^ (*N* − 2)/(1 − *R*^2^) [25].

### Bayesian colocalization analysis

To strengthen association evidence, we performed Bayesian colocalization analysis between mitochondrial-related cis-QTL meeting the above requirements (P_SMR-FDR correction < 0.05 and P _HEIDI > 0.05) and KOA using “coloc” package (version 5.2.3) in R software. And 1 Mb around the leading SNP was selected as colocalization region. The method of Bayesian colocalization can elucidate the possibility of shared genetic variation between two traits by presenting posterior probabilities for five hypotheses. In this study, we focused only on the posterior probabilities of Hypothesis 4 (PP.H4). Specifically, PP.H4 implied that the two traits were shared within the genetic region and were driven by the same variant. Here, we consider PP.H4 > 0.8 as the threshold for successful colocalization [26, 27].

### SMR-multi test

We further performed the SMR-multi test to strengthen the genetic association between the identified candidate mitochondrial-related genes and KOA as it considers all SNPs in a cis-QTL region [28]. First, all cis-eQTL SNPs that were located within 500 kb of the top-associated cis-eQTL and reached the *P* < 5 $\times$ 10^–8^ threshold were selected. Subsequently, those of them that passed the LD-square threshold (The default value is 0.1) were retained for final analysis. In addition, we checked the consistency of allele frequency of each SNP between pairwise datasets, including eQTL data, GWAS data, and LD reference data. SNPs with allele frequency differences < 0.20 between any pair of the data sets were included.

### Positive control study

To increase the credibility of the study and the SMR method, a positive control analysis was performed. Since the accelerating effect of Runt-related transcription factor 2 (RUNX2) on the development of OA is well-established [29–32], we thus investigated the association between RUNX2 expression levels and KOA as a positive control study for the SMR method.

## Results

### Mitochondrial-related gene expression and KOA

**Fig. 2:**
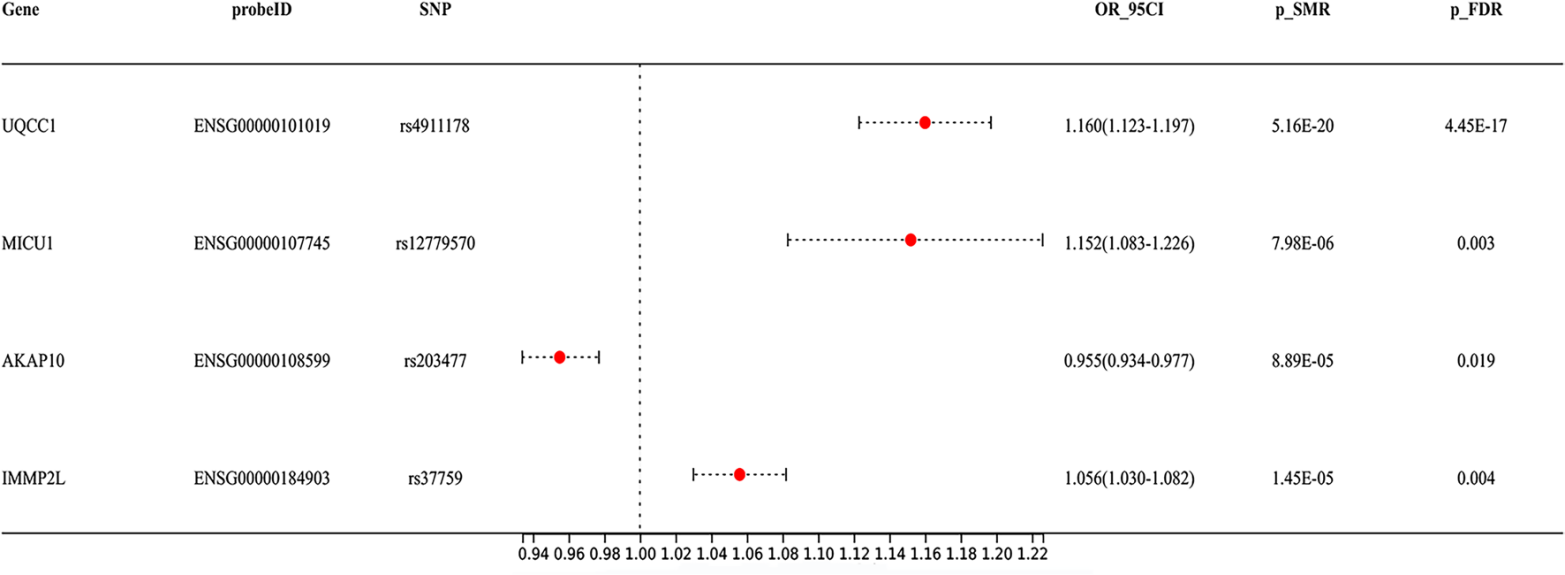
SMR results for the genetic association between mitochondrial gene expression and knee osteoarthritis is presented in forest plot

**Fig. 3:**
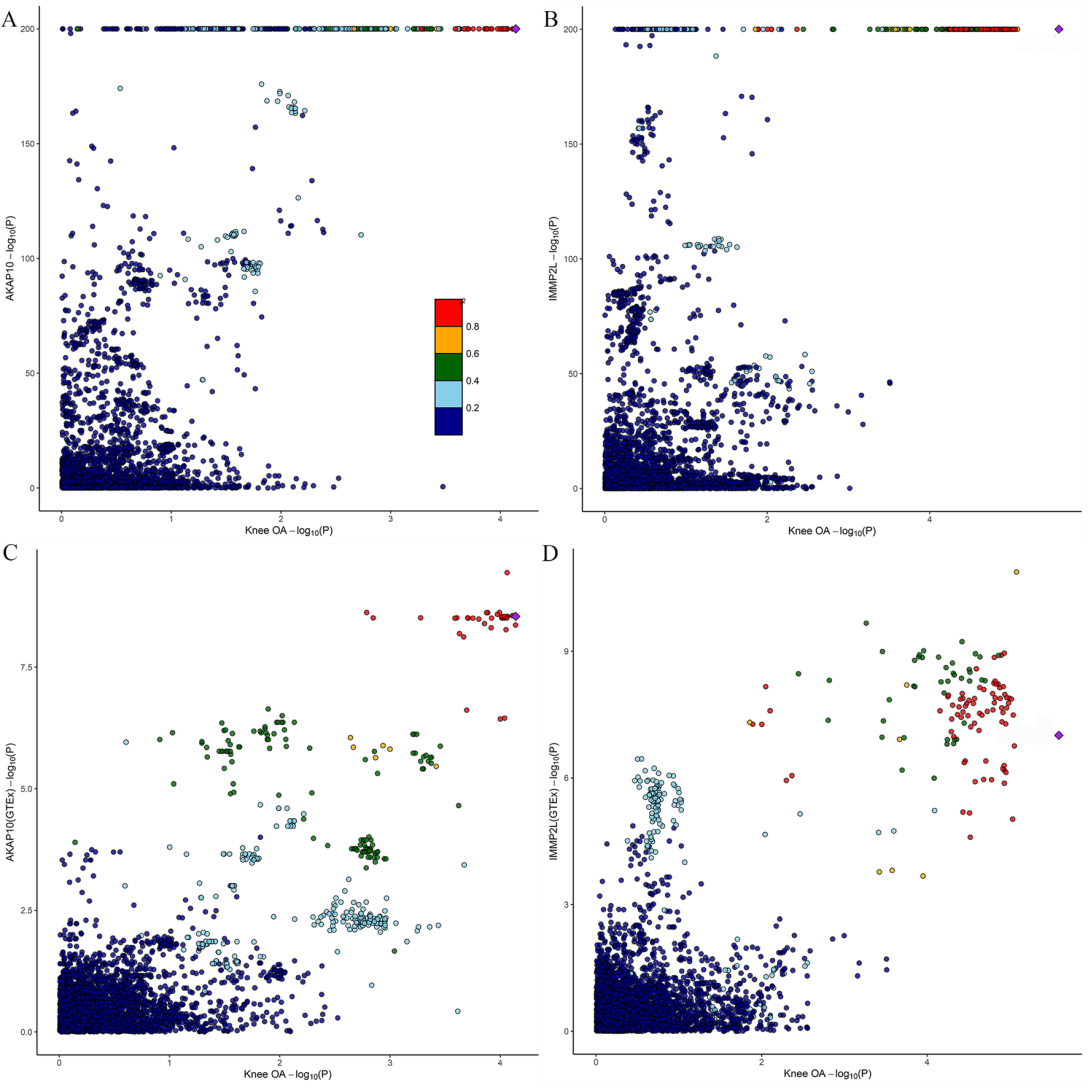
Locuscompare plot of Bayesian colocalization analysis for knee osteoarthritis and the expression of (**A**) AKAP10, (**B**) IMM2PL, (**C**) AKAP10 (GTEx consortium), and (**D**) IMM2PL (GTEx consortium)

We identified a total of 864 mitochondrial-associated genes from the eQTL consortium (Supplementary Table 2). After FDR correction, four genes were found to be associated with KOA risk. Specifically, elevated gene expression levels of ubiquinol-cytochrome c reductase complex assembly factor 1 (OR = 1.160; 95% CI, 1.123–1.197; *P-*_*FDR*_ = 4.45 $\times$ 10^–17^)(UQCC1), mitochondrial calcium uptake 1 (OR = 1.152; 95% CI, 1.083–1.226; *P-*_*FDR*_ = 0.003) (MICU1), and inner mitochondrial membrane peptidase subunit 2 (OR = 1.056; 95% CI, 1.030–1.082; *P-*_*FDR*_ = 0.004) (IMMP2L) increased the risk of KOA, whereas decreased gene expression levels of A-kinase anchoring protein 10 (AKAP10) increased the risk of KOA (OR = 0.955; 95% CI, 0.934–0.977; *P-*_*FDR*_ = 0.019) (Fig. 2). Notably, the F-statistics of the respective top SNPs selected for these four candidate genes are all over 10, indicating that our results are not affected by weak IVs (Supplementary Table 2). However, unfortunately, the association of UQCC1 and MICU1 (P _HEIDI < 0.05) with KOA was driven by pleiotropy (p_HEIDI test < 0.05) (Table 1). Furthermore, colocalization analysis demonstrated that AKAP10 (PP.H4 = 0.84) and IMMP2L (PP.H4 = 0.91) shared the same genetic variants with KOA (Table 1, Fig. 3), suggesting robust genetic association.

**Table 1:** Results of HEIDI test and colocalization analysis for the association between mitochondrial gene expression and knee osteoarthritis

Gene	SNP	Chr	Pos	p_HEIDI test	nsnp_HEIDI	PP.H4
UQCC1	rs4911178	20	33,952,620	0.002	20	
MICU1	rs12779570	10	74,177,771	0.001	20	
AKAP10	rs203477	17	19,831,819	0.461	20	0.84
IMMP2L	rs37759	7	110,934,682	0.186	20	0.91

*SNP* Single-nucleotide polymorphism, *HEIDI* Heterogeneity in the dependent Instruments, *PP.H4* Posterior probabilities of hypothesis 4

Consistent with our primary findings in discover study, our replication study similarly showed that genetically predicted expression levels of AKAP10 were associated with a decreased risk of KOA (OR = 0.962; 95% CI, 0.934–0.992; *P-value* = *0.012*), whereas expression levels of IMMP2L were associated with an increased risk of KOA (OR = 1.034; 95% CI, 1.001–1.068; *P-value* = 0.041) (Supplementary Table 4), which suggested the reliability of our results.

### Mitochondrial-related protein abundance and KOA

A total of 107 mitochondrial-associated proteins were matched to the GWAS summary data reported by Ferkingstad et al. However, no proteins were observed to be associated with the risk of KOA after FDR correction (Supplementary Table 3). This might be attributed to 1) incomplete development of pQTL data and 2) fewer proteins have cis-pQTLs that meet the requirements for performing SMR analysis.

### Mitochondrial-related gene expression in musculoskeletal tissues and KOA

Considering the tissue specificity of gene expression, we applied specific eQTL data from musculoskeletal tissues to strengthen the association between the identified candidate genes and KOA and reveal the deeper pathogenesis of KOA. Notably, AKAP10 (OR = 0.829; 95% CI, 0.743–0.926; *P* = 8.78 $\times$ 10^–04^) and IMMP2L (OR = 1.213; 95% CI, 1.096–1.343; *P* = 1.98 $\times$ 10^–04^) were also found to be associated with KOA in musculoskeletal tissues (Table 2). In addition, HEIDI test showed no pleiotropy between them (p_HEIDI test > 0.05) (Table 2). Further colocalization analyses were consistent with the primary findings (AKAP10 (PP.H4 = 0.81); IMMP2L (PP.H4 = 0.93)) (Table 2, Fig. 3).

**Table 2:** SMR results for association between musculoskeletal tissue-specific gene expression and knee osteoarthritis

Gene	Pos	SNP	OR (95CI)	p_SMR	p_HEIDI test	PP.H4
AKAP10(GTEx)	17	rs175922	0.829 (0.743–0.926)	8.78E-04	0.684	0.81
IMMP2L(GTEx)	7	rs214455	1.213 (1.096–1.343)	1.98E-04	0.210	0.93

*GTEx* Genotype-tissue expression, *SNP* Single-nucleotide polymorphism, *OR* Odds ratio, *CI* Confidence interval, *SMR* summary data-based Mendelian Randomization, *HEIDI* Heterogeneity in the dependent Instruments, *PP.H4* Posterior probabilities of hypothesis 4

### Results of SMR-multi test

A total of 1,879 eQTL SNPs significantly associated (*P* < 5 × 10^–8^) with AKAP10 expression and 1,687 eQTL SNPs significantly associated (*P* < 5 × 10^–8^) with IMMP2L expression were identified within their respective cis regions. However, only 59 cis-eQTL SNPs for AKAP10 and 51 cis-eQTL SNPs for IMMP2L passed the LD-square threshold 0.1 to be finally retained for SMR-multi test. As we expected, further SMR-multi test similarly showed that expression levels of IMMP2L were positively associated with the risk of KOA (*P-value* = 0.003), whereas expression levels of AKAP10 were negatively associated with the risk of KOA (*P-value* = 0.023) (Supplementary Table 5).

### Results of positive control study

Positive control study showed that high expression levels of RUNX2 were associated with an increased risk of KOA (OR = 1.040; 95% CI, 1.006–1.075; *P-value* = 0.021) (Supplementary Table 6), suggesting the efficacy of the SMR method. Subsequent HEIDI test suggested that the association of RUNX2 expression levels with KOA (p_HEIDI test = 0.494) was not driven by pleiotropy (Supplementary Table 6).

## Discussion

Using large-scale QTL data and GWAS summary data, we performed SMR analysis to examine the association of genetically proxied mitochondrial dysfunction with KOA risk. By combining SMR analysis and HEIDI test, the expression levels of 2 mitochondrial-related gene were found to be associated with KOA risk. Specifically, elevated gene expression levels of IMMP2L increased the risk of KOA, whereas increased gene expression levels of AKAP10 decreased the risk of KOA. Colocalization analysis demonstrated that AKAP10 and IMMP2L shared the same genetic variant with KOA. Additionally, consistent results were found in replication study, musculoskeletal tissues, and SMR-multi test, further suggesting the reliability of our findings and revealing the important role of mitochondrial dysfunction mediated by these genes in the pathogenesis of KOA.

AKAP10 gene, located on human chromosome 17, encodes a multi subunit protein that binds to the regulatory subunit of protein kinase A (PKA) [33]. Previous studies confirmed its important role in humans [34]. In recent decades, genetic variants of AKAP10 have been associated with cardiac arrhythmias[35], breast cancer[36], and preterm birth [37]. However, limited reports exist on the relationship between AKAP10 and OA. In the present study, we found that increased AKAP10 expression was associated with a reduced risk of KOA. The association can be reasonably explained through two possible mechanisms. First, AKAP10 contributes to the cholinergic/vagal signaling pathway and increases vagal sensitivity [35]. Notably, the presence of cholinergic nerves in most joint tissues, such as cartilage and subchondral bone, has been well described in a systematic review [38]. Some studies have found that vagus nerve activation can inhibit the production of cytokines (e.g., tumor necrosis factor (TNF), which may ameliorate joint inflammation [39]. Nicotine, an exogenous stimulator of the cholinergic system, has been shown to alleviate OA pain and slow cartilage degradation induced by sodium iodoacetate in mice [40]. Several in vitro tests have suggested that treatment with acetylcholinesterase inhibitors prevents cartilage degeneration and inflammation [41, 42]. Second, Kim et al. demonstrated that AKAP10 increased lipopolysaccharide-induced nitric oxide (NO) production [43]. NO, present in almost all types of human cells, has been shown to potentially have a protective effect on chondrocytes [44]. Surprisingly, NO was found to potentially relieve OA-related pain through blood flow, neurotransmitter pathways, opioid receptor pathways, and anti-inflammatory pathways [45]. Early subchondral bone loss and advanced subchondral bone sclerosis are important pathologic characteristics of OA [46, 47]. Therefore, the inhibition of excessive bone resorption mediated by osteoclasts in the early stages and abnormal bone formation mediated by osteoblasts in the late stages are currently the main therapeutic strategies of interest [48]. An older study by MacIntyre et al. revealed that NO inhibits the proliferation of osteoclasts and bone resorption in mice [49]. Many subsequent studies have found similar results [50, 51]. Notably, high concentrations of NO produced by NO donors or pro-inflammatory factors have been shown to be effective in inhibiting osteoblast growth and differentiation [52, 53]. Taken together, AKAP10 may slow the progression of OA by promoting the cholinergic/vagal nervous system and NO production.

IMMP2L encodes a protein responsible for cleaving signal peptide sequences of cytochrome c1 (CYC1) and mitochondrial glycerophosphate dehydrogenase 2 (GPD2) [54]. IMMP2L has previously been investigated in relation to ovarian aging [55] and autism[56]; however, limited research has been conducted on its association with KOA. Our results provide evidence that IMMP2L is a risk factor for KOA. Consistent with our findings, Lawther et al. recently observed decreased ROS in IMMP2L knockout mice [57]. Considering the close relationship between KOA and ROS, this finding appears plausible. More mechanisms underlying IMMP2L-induced KOA remain to be investigated.

The primary strength of our study is that we used a MR design, which diminished the bias resulting from confounders and reverse causality of traditional observational studies. In addition, evidence for genetic association was further strengthened by the success of colocalization analysis and HEIDI test. More importantly, consistent results were observed in replication study, musculoskeletal tissues, and SMR-multi test, suggesting the reliability of our findings. However, there are still several limitations. First, although the GWAS summary data for KOA in discover study we used were largest available, participants involved in this study were not exclusively of European ethnicity. It may lead to some bias. Surprisingly, fewer than 3% of the participants were of non-European ancestry; thus, we believe that the results remain robust. Second, there was a certain unmeasurable sample overlap that existed between the KOA GWAS datasets used in the primary and replication studies. It is caused by the inherent limitations of the development and updating of GWASs, as majority of the current large OA GWASs have been expanded and updated by adding new cohorts to the original data [22, 23, 58, 59]. Thus, we had to acknowledge this limitation that cannot be mitigated. In addition, probably due to the pQTL data have not been fully exploited yet, we failed to investigate any mitochondrial-related proteins associated with KOA. We expect more researchers to follow up with further exploration of pQTL data. Furthermore, the candidate genes we identified for KOA have small effect size and need to be carefully considered in clinical application. Finally, there were no available independent GWAS summary data that directly represent mitochondrial dysfunction, so we used genetically predicted mitochondrial-related genes to represent it.

## Conclusion

In summary, our SMR and colocalization analyses examined the genetic association between mitochondrial dysfunction proxied by mitochondrial-related genes and KOA and revealed the important role of mitochondrial dysfunction mediated by these genes in the pathogenesis of KOA. Furthermore, these identified candidate genes offer the possibility of clinical drug target development for KOA. Further research was needed to validate our findings.

## Supplementary Information

Below is the link to the electronic supplementary material.

Supplementary file1 (DOCX 16 KB)

Supplementary file2 (XLSX 241 KB)

Supplementary file3 (XLSX 38 KB)

Supplementary file4 (XLSX 10 KB)

Supplementary file5 (XLSX 9 KB)

Supplementary file6 (XLSX 9 KB)

## Data Availability

1136 mitochondrial-related genes were extracted from the human MitoCarta3.0 database (https://personal.broadinstitute.org/scalvo/MitoCarta3.0/human.mitocarta3.0.html). The GWAS summary data for KOA were obtained from Genetics of Osteoarthritis Consortium (Discovery study: https://msk.hugeamp.org/downloads.html) and UK Biobank and arcOGEN Consortium (Replication study: https://www.ebi.ac.uk/gwas/publications/30664745), respectively. The blood eQTL data were download from the eQTLGen Consortium (https://www.eqtlgen.org/cis-eqtls.html). The blood pQTL data were download from the DeCODE genetics (https://download.decode.is/form/folder/proteomics). The musculoskeletal tissues eQTL data were download from the SMR tools software (https://yanglab.westlake.edu.cn/data/SMR/GTEx_V8_cis_eqtl_summary.html).
