# Supplementary material for: Identification of genetic association between mitochondrial dysfunction and knee osteoarthritis through integrating multi-omics: a summary data-based Mendelian randomization study
Source: Clin Rheumatol. 2024 Sep 11;43(11):3487–96. doi: 10.1007/s10067-024-07136-7 (PMC11489259; doi:10.1007/s10067-024-07136-7)
Supplement: Supplementary file 1 — Supplementary file1 (DOCX 16 KB) [file 10067_2024_7136_MOESM1_ESM.docx]

| Data subtype | Data | Sample size | Population | Author | Resource |
| --- | --- | --- | --- | --- | --- |
| QTL | Blood cis-eQTL | 31,684 | European | Võsa U, et al. | eQTLGen Consortium |
| QTL | Blood cis-pQTL | 35,559 | European | Ferkingstad E et al. | DeCODE genetics |
| QTL | Musculoskeletal cis-eQTL | 706 | European |  | GTEx Consortium |
| GWAS summary | Knee osteoarthritis (Discover) | 396,054 | Mix | Boer CG et al. | Genetics of Osteoarthritis Consortium |
| GWAS summary | Knee osteoarthritis (Replication) | 403,124 | European | Tachmazidou I et al. | UK Biobank and arcOGEN Consortium |

**Supplementary Table 1** Details of data resources

QTL, quantitative trait loci; GWAS, genome-wide association studies; arcOGEN, Arthritis Research UK Osteoarthritis Genetics (arcOGEN)
